# Electroconvulsive therapy and informed consent in compulsory treatment – an ethical dilemma

**DOI:** 10.1192/j.eurpsy.2021.1892

**Published:** 2021-08-13

**Authors:** C. Cunha, R. Pereira, G. França, J. Silva

**Affiliations:** Psychiatry Department, Magalhães Lemos Hospital, Porto, Portugal

**Keywords:** Electroconvulsive therapy, compulsory treatment, mental health legislation, informed consent

## Abstract

**Introduction:**

Given the effectiveness and overall safety in several psychiatry conditions, electroconvulsive therapy remains a widely used procedure in current medical practice. Informed consent is still a requirement for the use of ECT both in voluntary and compulsory treatment; however, since severe mental illness can affect decision-making capacity and insight of the need for treatment, this requirement often constitutes an obstacle to its use. In addition, stigma around ECT still contributes to treatment refusal.

**Objectives:**

To summarize the most recent evidence published about ECT and discuss the ethical and legal implications of its use, enlightened by the empirical description of a clinical vignette.

**Methods:**

Review of literature on the ethical and legal issues involving the ECT use in patients on compulsory treatment, considering the efficacy, risks, the mental health legislation in Portugal, and several international directives.

**Results:**

Informed consent is the basic tenet in the contemporary physician-patient relationship. In principle, ECT can only be administered to patients who prior consent to the treatment. In contemporary practice, providing the best medical assistance and respecting the patient’s autonomy are two fundamental principles. However, we often face an ethical dilemma, when severely ill patients, whose insight, the ability for self-determination and decision-making capacity may be impaired, refuse a potential beneficial treatment as ECT.

**Conclusions:**

The use of ECT in severe mental illness is still hampered by legal and ethical constraints. A future revision of the law could protect patients from being excluded from a treatment that may change the course of the disease.

**Disclosure:**

No significant relationships.

